# Rap2B promotes proliferation, migration, and invasion of human breast cancer through calcium-related ERK1/2 signaling pathway

**DOI:** 10.1038/srep12363

**Published:** 2015-07-23

**Authors:** Jiehui Di, Hui Huang, Debao Qu, Juangjuan Tang, Wenjia Cao, Zheng Lu, Qian Cheng, Jing Yang, Jin Bai, Yanping Zhang, Junnian Zheng

**Affiliations:** 1 Cancer Institute, Xuzhou Medical College, Xuzhou 221002, Jiangsu, P.R. China; 2 Jiangsu Center for the Collaboration and Innovation of Cancer Biotherapy, Cancer Institute, Xuzhou Medical College, Xuzhou 221002, Jiangsu, P.R. China; 3 Department of Oncology, the People’s Hospital of Kaixian, Kaixian 405400, Chongqing, P.R. China; 4 Department of Radiation Oncology and Lineberger Comprehensive Cancer Center, School of Medicine, the University of North Carolina at Chapel Hill, 101 Manning Drive, Chapel Hill, NC 27514, USA

## Abstract

Rap2B, a member of GTP-binding proteins, is widely upregulated in many types of tumors and promotes migration and invasion of human suprarenal epithelioma. However, the function of Rap2B in breast cancer is unknown. Expression of Rap2B was examined in breast cancer cell lines and human normal breast cell line using Western blot analysis. Using the CCK-8 cell proliferation assay, cell cycle analysis, and transwell migration assay, we also elucidated the role of Rap2B in breast cancer cell proliferation, migration, and invasion. Results showed that the expression of Rap2B is higher in tumor cells than in normal cells. Flow cytometry and Western blot analysis revealed that Rap2B elevates the intracellular calcium level and further promotes extracellular signal-related kinase (ERK) 1/2 phosphorylation. By contrast, calcium chelator BAPTM/AM and MEK inhibitor (U0126) can reverse Rap2B-induced ERK1/2 phosphorylation. Furthermore, Rap2B knockdown inhibits cell proliferation, migration, and invasion abilities via calcium related-ERK1/2 signaling. In addition, overexpression of Rap2B promotes cell proliferation, migration and invasion abilities, which could be neutralized by BAPTM/AM and U0126. Taken together, these findings shed light on Rap2B as a therapeutic target for breast cancer.

Breast cancer is by far the most frequently diagnosed cancer and the second leading cause of cancer death in women worldwide^1^. Unfortunately, for all breast cancer patients, the long-term recurrent rates can be as high as 40%, of which 10%–20% are local and 60%–70% are distant metastases^2^. Tumor metastasis is a complex process mainly involving cell proliferation, migration, invasion, adhesion and vessel formation^3^. Therefore, unraveling the molecular mechanisms underlying breast cancer progression and metastasis would reveal lead molecules for targeted therapy^4^.

The Rap family of small GTP-binding proteins is composed of five members, namely, Rap1A, Rap1B, Rap2A, Rap2B and Rap2C, which are grouped into two subfamilies, namely, Rap1 and Rap2^5^. Studies have indicated that Ras family members are implicated in a range of biological functions in human cells, such as signal transduction, proliferation and migration^6^,^7^. Rap1 has elicited much stronger interest than the highly homologous Rap2 proteins; however, the role of Rap1 in carcinogenesis remains controversial. On one hand, aberrant activation of Rap1 leads to increased cancer cell proliferation and carcinogenesis^6^,^8^; on the other hand, inactivation of the Rap1 promotes invasion of osteosarcoma cells^9^. Although the effector region of Rap2 proteins differs from that of Rap1 proteins by just one residue, the exact role of Rap2 in carcinogenesis remains obscure.

Rap2B was first discovered from platelet cDNA library in the early 1990s^10^,^11^. Rap2B, being one of the members of the Ras superfamily, was predominantly upregulated in many types of tumors^12^. Renewed interest in Rap2B as a novel candidate oncogene in lung cancer rapidly mounted. Increased level of Rap2B expression is observed in lung cancer, and is involved in tumorigenesis through activation of the NF-kappa B pathway^13^. Subsequently, foci formation assay and wound-healing assay revealed that the extrinsic expression of Rap2B could transform NIH3T3 cell^14^. In addition, Rap2B as a novel p53 target participates in p53-mediated pro-survival function, which also raises the possibility that targeting Rap2B could sensitize tumor cells to apoptosis in response to DNA damage^12^. A recent study has reported that miR-342-3p targets Rap2B to suppress cell proliferation, migration, and invasion of non-small cell lung cancer^15^. Previously, we have demonstrated that Rap2B promotes migration and invasion of human suprarenal epithelioma. However, the expression and function of Rap2B have not been fully elucidated in the development of human breast cancer.

In the current study, we showed that the expression level of Rap2B was higher in breast cancer cells than in normal cells. Furthermore, Rap2B could upregulate the intracellular calcium level and the phosphorylation level of extracellular signal-related kinase (ERK) 1/2, which could be weakened by the cell-permeable calcium chelator BAPTM/AM and the specific inhibitors of MEK1/2 (U0126). Moreover, we also identified that Rap2B increased cell proliferation, migration and invasion abilities by upregulating calcium-related ERK1/2 signaling pathway. Our study may provide a potential therapeutic target for human breast cancer.

## Results

### Rap2B expression is increased in breast cancer

**Figure 1:**
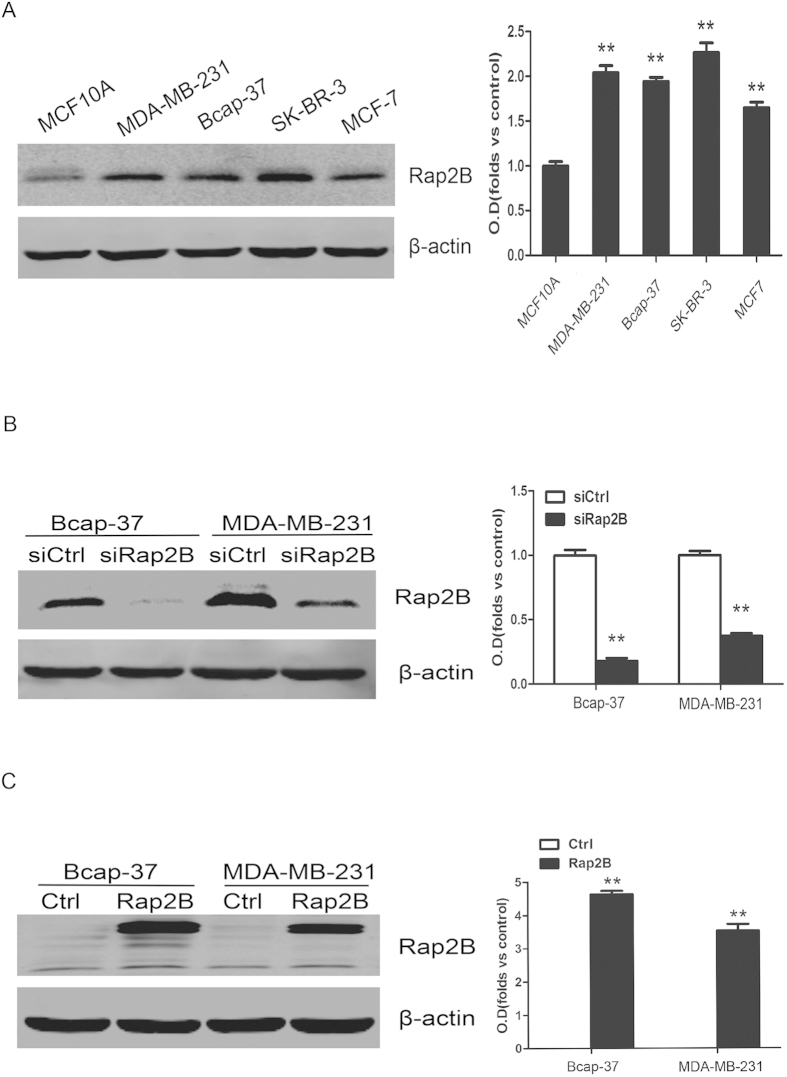
Protein expression level of Rap2B in breast cancer cell lines. (**A**) Western blot analysis of Rap2B expression in MCF-10A, MDA-MB-231, Bcap-37, SK-BR-3 and MCF-7 group with Rap2B antibody. β-actin served as loading control. The intensity of Rap2B was quantified by densitometry (software: Image J, NIH). (**B**) Western blot analysis of Rap2B in the Rap2B knockdown and control siRNA group for both Bcap-37 and MDA-MB-231 cell lines. The intensity of Rap2B was quantified by densitometry. (**C**) Western blot analysis to measure the protein levels of Rap2B in breast cancer cells transfected with Rap2B plasmids and pcDNA3.1-Myc3 plasmids. The intensity of Rap2B was quantified by densitometry. All experiments were carried out in triplicate. Data are presented as mean ± SD (n = 3). **P < 0.01 in comparison with respective group.

To investigate whether different expressions of Rap2B exist in breast cancer development, Western blot assay was performed using breast cancer cell lines and human normal breast epithelial cell line, MCF10A. It was clear that the breast cancer cell lines had significant increase expression as compared with MCF10A (Fig. 1A). These results showed that Rap2B is upregulated in breast cancer. Small interfering RNA (siRNA) was used to knockdown Rap2B expression in both Bcap-37 and MDA-MB-231 cells. Forty-eight hours after transfection, Rap2B protein was drastically decreased (Fig. 1B). In addition, pcDNA3.1-Myc3 control or pcDNA3.1-Myc3-Rap2B plasmids were transiently transfected into both cell lines. Twenty-four hours after transfection, Rap2B protein was significantly overexpressed (Fig. 1C).

### Rap2B regulates cell proliferation in breast cancer cells

**Figure 2:**
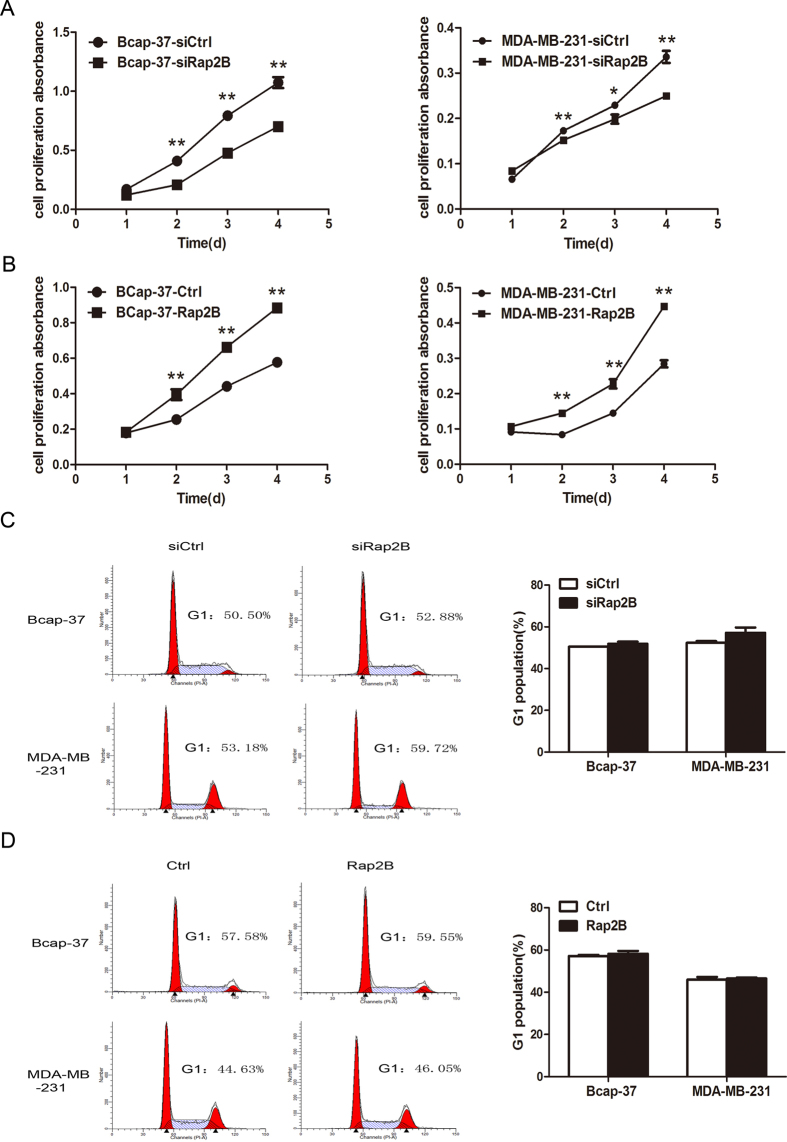
Effect of Rap2B on cell proliferation of breast cancer cells. (**A**) CCK-8 cell proliferation assay after Rap2B knockdown in Bcap-37 and MDA-MB-231 cells. (**B**) CCK-8 cell proliferation assay after Rap2B overexpressed in breast cancer cells. (**C**) Influence of Rap2B gene silencing on the cell cycle of breast cancer cells. The percentage of G1 population cells was measured by flow cytometry after Rap2B knockdown in breast cancer cells. (**D**) Influence of Rap2B gene overexpression on cell cycle analysis of breast cancer cells. The percentage of cells at G1 stage was calculated using ModFit LT 3.0 software. All experiments were carried out in triplicate. Data are presented as mean ± SD (n = 3). *P < 0.05, **P < 0.01 in comparison with respective group.

To further validate whether Rap2B affects carcinoma cell survival, cell viability was determined by CCK-8 cell proliferation assay. Knockdown of Rap2B inhibited cell growth in both Bcap-37 and MDA-MB-231 cells compared with the cells transfected with the negative control siRNA (Fig. 2A). At the same time, our data demonstrated that overexpression of Rap2B increased cellular growth in both breast cancer cell lines (Fig. 2B). To evaluate whether the reduced cell proliferation resulting from Rap2B knockdown was due to cell cycle arrest, we subsequently performed flow cytometry analysis to determine the role of Rap2B in cell cycle distribution. As expected, knockdown or restoration of Rap2B has no effect on cell cycle distribution of breast cancer cells (Fig. 2C,D). The data above indicated that Rap2B could promote cell proliferation, but has no effect on the cell cycle.

### Rap2B induces calcium-dependent phosphorylation of ERK

**Figure 3:**
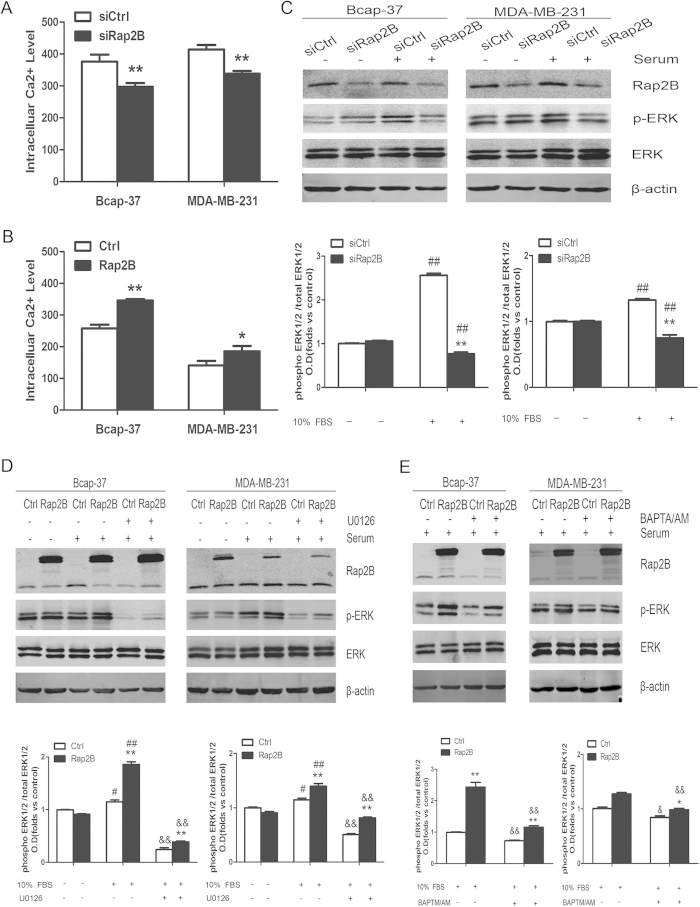
Rap2B can increase the intracelluar calcium level and induce ERK1/2 phosphorylation. (**A**) Rap2B siRNA could decrease intracelluar calcium level in Bcap-37 and MDA-MB-231 cell lines, as measured by a flow cytometer. (**B**) Rap2B overexpression increased intracelluar calcium level in breast cancer cells. (**C**) After breast cancer cells were transfected and starved, some cells were treated with 10% FBS and some were not. Western blot analysis of the protein levels of Rap2B, p-ERK, and ERK in Rap2B knockdown and control siRNA group for both cell lines. (**D**) After transfection and starvation, cells were incubated in the presence or absence of U0126 for 30 min. Then, some cells were treated with 10% FBS and some were not. Western blot analysis of the protein levels of Rap2B, p-ERK, and ERK in Rap2B overexpression and control group for both cell lines. (**E**) After transfection and starvation, cells were incubated in the presence or absence of BAPTA/AM for 30 min. Then, some cells were treated with 10% FBS. Western blot analysis of the protein levels of Rap2B, p-ERK, and ERK in Rap2B over-expression and control group for both cell lines. All experiments were carried out in triplicate. Data are presented as mean ± SD (n = 3). *P < 0.05, **P < 0.01 in comparison to respective group, ^#^*P* < 0.05, ^##^*P* < 0.01 in comparison with respective 10% FBS-untreated group, and ^&^*P* < 0.05, ^&&^*P* < 0.01 in comparison with respective inhibitor-untreated group.

Stope *et al.* reported that Rap2B directly interacts with and induces activation of PLCε^16^. PLCε catalyzes the hydrolysis of phosphatidylinositol 4, 5-bisphosphate, and strongly increases diacylglycerol (DAG) accumulation and inositol 1,4,5-trisphosphate (IP_3_) formation. Subsequently, IP_3_ triggers calcium mobilization resulting in rapid increase in intracellular calcium concentrations. Calcium ion is an intracellular second messenger that regulates numerous biological processes^17^,^18^. Thus, we performed flow cytometry to determine the intracellular calcium level in breast cancer cells. Data revealed that intracellular calcium level is suppressed after knockdown of Rap2B in Bcap-37 and MDA-MB-231 cells, whereas overexpression of Rap2B upregulates intracellular calcium level (Fig. 3A,B). Then we performed Western blot analysis to detect the total expression and phosphorylation levels of ERK1/2 in breast cancer cells, which is an important downstream substrate for calcium ions. After transfection and starvation, serum was used to increase phosphorylation of ERK1/2 for 30 min in both Bcap-37 and MDA-MB-231 cells. Inhibition of ERK1/2 phosphorylation was observed after Rap2B knockdown in breast cancer cells (Fig. 3C), whereas the total expression of ERK1/2 did not change. Meanwhile, our data also showed that the phosphorylation level of ERK1/2 increased in both cell lines transfected with Rap2B (Fig. 3D–F). In addition, the cell-permeable calcium chelator BAPTM/AM and the specific inhibitor of MEK1/2 (U0126) were used to study their effects in Rap2B-mediated signaling. The upregulation of phosphorylated ERK1/2 induced by Rap2B overexpression was aborted by U0126 and BAPTM/AM in both cell lines (Fig. 3D,E). However, Rap2B expression did not change after treatment with those inhibitors (Fig. 3D–F). These results indicated that Rap2B is an upstream regulator of the calcium-related ERK1/2 signaling pathway.

### Rap2B could promote cell migration and invasion via Rap2B-calcium-ERK1/2 signaling pathway

**Figure 4:**
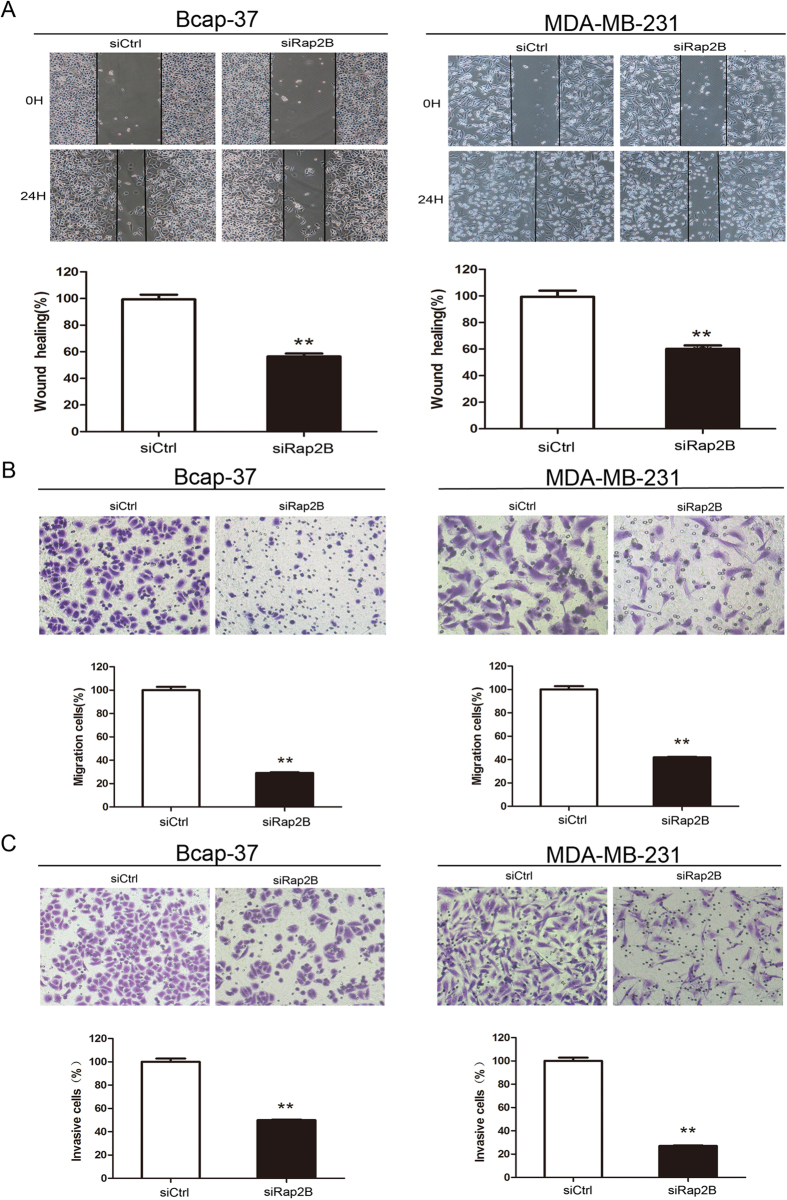
Knockdown of Rap2B inhibits breast cancer cell migration and invasion abilities. (**A**) Wound-healing assay was performed after Rap2B knockdown in Bcap-37 and MDA-MB-231 cell lines. (**B**) Cell migration assay was carried out after Rap2B knockdown in breast cancer cells. (**C**) A matrigel cell invasion assay was carried out after Rap2B knockdown in breast cancer cells. All experiments were carried out in triplicate. Data are presented as mean ± SD (n = 3). **P < 0.01 in comparison with respective group.

**Figure 5:**
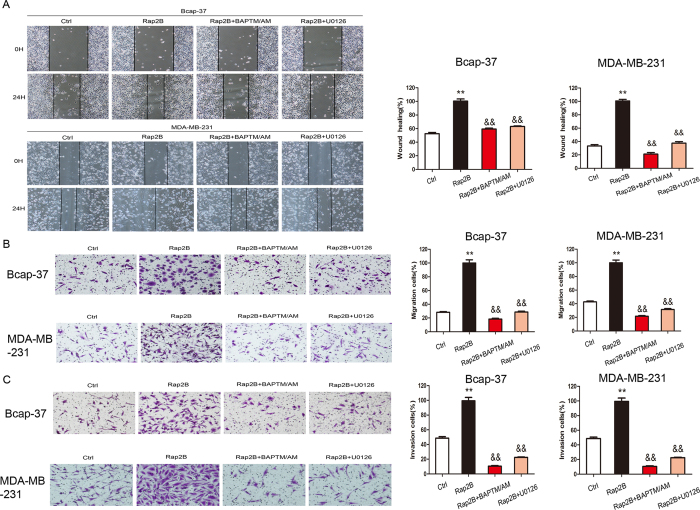
Rap2B overexpression promotes breast cancer cell migration and invasion abilities. (**A**)Wound-healing assay was performed after reintroduction of Rap2B in Ctrl, Rap2B, Rap2B + BAPTA/AM and Rap2B + U0126 group in Bcap-37 and MDA-MB-231 cell lines. Starved cells were incubated with 30 μmol/L BAPTA/AM and 10 μmol/L U0126. (**B**) Cell migration assay was carried out after overexpression of Rap2B in Ctrl, Rap2B, Rap2B+BAPTA/AM and Rap2B+U0126 group in breast cancer cells. Starved cells were incubated with 30 μmol/L BAPTA/AM and 10 μmol/L U0126. (**C**) A matrigel cell invasion assay was carried out after overexpression of Rap2B in Ctrl, Rap2B, Rap2B + BAPTA/AM and Rap2B + U0126 group in breast cancer cells. Starved cells were incubated with 30 μmol/L BAPTA/AM and 10 μmol/L U0126. All experiments were carried out in triplicate. Data are presented as mean ± SD (n = 3). **P < 0.01 in comparison with respective group, and ^&^*P* < 0.05, ^&&^*P *< 0.05 in comparison with respective inhibitor-untreated group.

Rap2B could upregulate cell proliferation and induce calcium-dependent phosphorylation of ERK, so we sought to determine whether Rap2B could affect cell migration and invasion. Wound-healing assay, migration assay and matrigel invasion assay were carried out in breast cancer cells. In cell wound-healing assay, 10 μg/ml mitomycin C was incubated for 2 h prior to the scratch assay, which inhibited mitosis of the cells and allowed us to distinguish migration from proliferation^19^,^20^,^21^,^22^. Data showed that the wound-healing rate in Bcap-37 and MDA-MB-231 cells with Rap2B knockdown is slower than that in control siRNA group (Fig. 4A). In cell migration assay, we found that Rap2B knockdown suppresses cell migration ability by 71% and 58% in Bcap-37 and MDA-MB-231 cells, respectively (Fig. 4B). In cell invasion assay, silencing of Rap2B inhibits cell invasive ability of Bcap-37 and MDA-MB-231 cells by 50% and 73%, respectively (Fig. 4C). Meanwhile, restoration of Rap2B promotes cell migration and invasion in breast cancer cells *in vitro* (Fig. 5A–C). Collectively, results illustrate that Rap2B could enhance the migration and invasion of Bcap-37 and MDA-MB-231 cells *in vitro*.

Thus, we inferred that Rap2B promotes migration and invasion of breast cancer cells via calcium related-ERK1/2 signaling pathway. To further validate our assumption, we investigated the involvement of calcium and ERK1/2 on the Rap2B-induced migratory effect in migration and invasion assays using specific pathway inhibitors, namely, BAPTM/AM (30 μM) and U0126 (10 μM), respectively. As expected, both pharmacological inhibitors reduced Rap2B-induced effect on cell migration and invasion of breast cancer cells (Fig. 5A–C). These data collectively showed that Rap2B overexpression increases the level of calcium, which subsequently mediates calcium related-ERK1/2 signaling pathway activation, and finally leads to breast cancer cell migration and invasion.

## Discussion

Rap2B gene was originally cloned from the cDNA library of human platelets and is located at 3q25.2 of human chromosome, which is a hotspot of cancer research^23^,^24^. Rap2B protein is a member of the Ras superfamily of small GTPases. Studies have shown that Ras gene mutations or overexpressions are involved in oncogenesis of a variety of human tumors and in poor prognostic significance for survival^25^. Aberrant activation of Rap is implicated in processes such as cell cycle control, migration and invasion of cancer cells^6^,^8^,^26^,^27^. Rap2 protein expression has been detected to be several folds higher in human thyroid cancer cells^28^. Notably, Rap2 reportedly induces cytoskeleton rearrangements and promotes cell rounding and migration^29^,^30^. Moreover, Rap2A is involved in tumor formation and malignant progression in human prostate and follicular thyroid cancers^31^,^32^. A new study has shown that Rap2B supports the oncogenic status and acts as an oncogene in human cancers^12^,^15^. In this study, our results demonstrated that Rap2B expression is predominantly increased in breast cancer cell lines (Fig. 1A), which is consistent with the result of our previous study indicating that the levels of Rap2B expression are all significantly higher in tumor tissues than those in adjacent, normal renal tissues^33^. Taken together, we infer that Rap2B might play an important role in the development of breast cancer.

Schmidt *et al.* first reported that Rap2B stimulates PLCε and induces calcium increase^34^,^35^. Active Rap2B strongly enhances the formation of diacylglycerol [DAG] and inositol 1,4,5-trisphosphate [IP3], and the subsequent activation of protein kinase C isoforms and Ca^2+^ release from intracellular stores^36^. Our present study also confirmed that intracellular calcium level is suppressed after knockdown of Rap2B, whereas it is enhanced by Rap2B overexpression (Fig. 3A,B). However, the mechanism through which Rap2B regulates calcium release in breast cancer cells remains to be elucidated. Calcium could transfer the signal to the downstream signaling molecules leading to ERK1/2 activation. ERK, which is a member of the mitogen-activated protein kinase pathway, is reported to induce cell-cycle signaling to promote cell proliferation and differentiation^37^. We found that the ability of cell proliferation is decreased after Rap2B knockdown in breast cancer cells, and high proliferative activity of tumor cells is associated with increased cell-cycle transition^38^. Our data showed that knockdown of Rap2B does not arrest cell-cycle progression at the G1 to S transition (Fig. 2C,D), which is similar to the findings of Zhang^12^. Our data also showed that increased Rap2B enhances the expression of phosphorylated ERK1/2, whereas the total levels remain unchanged (Fig. 3D). At the same time, ERK1/2 phosphorylation induced by Rap2B was blocked by the cell-permeable calcium chelator (BAPTM/AM) and by the specific inhibitor of ERK1/2 (U0126) (Fig. 3D–F). However, Rap2B expression did not change after treatment with those inhibitors (Fig. 3D–F). These results suggested that Rap2B should be an upstream factor modulating the Ca^2+^-related ERK1/2 signaling pathways in breast cancer.

Calcium is an ubiquitous second messenger that is known to be involved in several fundamental physiological functions, such as cell cycle control, survival, migration, and gene expressions^39^,^40^. ERK, which is a known effector for cytoskeletal regulation, promotes epithelial-mesenchymal transition and facilitates cell migration, such as of pancreatic cancer cells^41^. Given that Rap2B is an upstream target of the Ca^2+^-related ERK1/2 signaling pathway in cancer cells, Rap2B possibly contributes to important events during tumor progression, such as cell proliferation, migration, invasion, and metastasis^42^,^43^. Tissue invasion and metastasis is an exceedingly complex process that represents one of the six initial cancer hallmarks^44^. Our present study first demonstrated that significant promotions of invasion and migration by overexpression of Rap2B exist in breast cancer cell lines (Fig. 5). As expected, migration and invasion abilities can be suppressed by inhibitors BAPTM/AM and U0126, respectively. This result is similar to that of Wang *et al.*^45^ which suggests that calcium-related ERK1/2 signaling positively mediates Rap2B-stimulated cell growth, migration and invasion. Our previously published paper demonstrated that Rap2B can promote migration and invasion of human suprarenal epithelioma by increasing MMP-2 protein expression and activity. We also examined the levels of MMP-2 and MMP-9 in the present study, and our data demonstrated that knockdown of Rap2B has no effect on protein expression and enzyme activity of MMP-2 and MMP-9.

**Figure 6:**
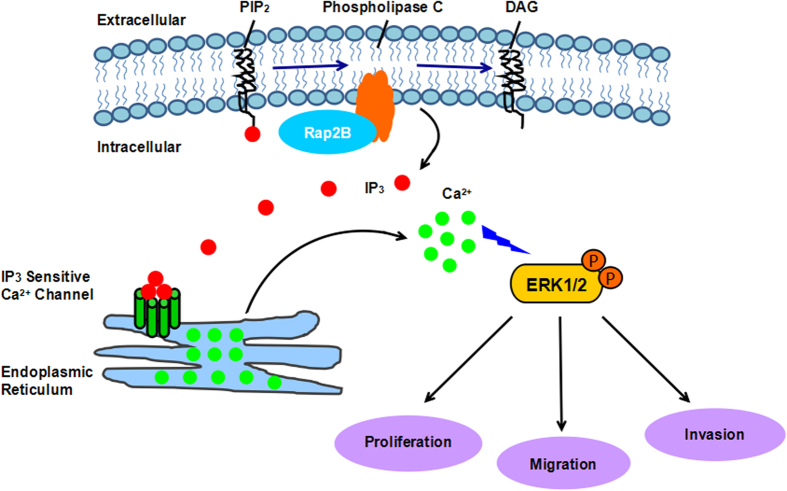
A schematic diagram illustrating the proposed Rap2B-induced calcium-related ERK1/2 signaling pathway involved in breast cancer cell proliferation, migration, and invasion. Rap2B increases the intracellular calcium level, which subsequently phosphorylates the downstream target of ERK1/2, thereby promoting breast cancer cell proliferation, migration, and invasion *in vitro*. Together, it will support the novel notion that Rap2B promotes tumor cell proliferation, migration, and invasion via calcium-related ERK1/2 signaling pathway.

In summary, our results showed that Rap2B is expressed at high levels in breast cancer cell lines, and that Rap2B could induce calcium-dependent phosphorylation of ERK1/2. Furthermore, we demonstrated that Rap2B can influence breast cancer cell proliferation, migration, and invasion through elevated intracellular calcium level and further promote ERK1/2 phosphorylation, as shown in Fig. 6. Further experiments are underway to evaluate the effect of Rap2B on cell migration and invasion by regulating Ca^2+^-related ERK1/2 signaling pathway using an *in vivo* tumor mouse model, hoping that we can provide a useful therapeutic strategy to help restrain the progression of breast cancer.

## Materials and Methods

### Cell culture and transfection

Human breast carcinoma cell lines (Bcap-37, MDA-MB-231, SK-BR-3 and MCF-7) and human breast epithelial cell line (MCF10A) were purchased from the Shanghai Institute of Biochemistry and Cell Biology, Chinese Academy of Sciences (Shanghai, China). Bcap-37 cells were grown in RPMI 1640 medium, MDA-MB-231 cells were maintained in L15 medium, and SK-BR-3 and MCF-7 were cultured in DMEM medium. All these media were supplemented with 10% fetal bovine serum (Invitrogen, Shanghai, China). MCF10A cells were cultured in DMEM/F12 medium. Nonspecific control siRNA or Rap2B siRNA (Invitrogen, Shanghai, China) was transfected into Bcap-37 and MDA-MB-231 cells at 50% confluence by siLentFect Lipid Reagent (Bio-Rad, Hercules, CA) according to the manufacturer’s protocol. The pcDNA3-Myc3-control and pcDNA3-Myc3-Rap2B expression plasmids were obtained from Dr. Yanping Zhang (The University of North Carolina at Chapel Hill, North Carolina, USA). Cells were grown to 90% confluence before being transiently transfected with Rap2B plasmids using Lipofectamine 2000 (Invitrogen, Shanghai, China) according to the manufacturer’s protocol.

### Western blot analysis

After performing specific treatments, cells were harvested and washed three times with PBS. After insoluble debris was pelleted by centrifugation at 15,000 *g* for 15 min at 4 °C, the supernatants were collected and examined for protein concentrations using the bicinchoninic acid (BCA) kit (Pierce, USA) according to the manufacturer’s instructions. Proteins were separated by electrophoresis on SDS-polyacrylamide gel electrophoresis (PAGE) and electro-transferred to nitrocellulose filter (NC) membrane. After being blocked for 2 h in 5% bovine serum albumin (BSA), membranes were incubated overnight at 4 °C with the following primary antibodies: anti-Rap2B, anti-p-ERK1/2, anti-ERK1/2 and anti-β-actin. Membranes were then washed and incubated with secondary antibodies conjugated with IRDye 680 or IRDye 800 (Rockland). Fluorescent signals were visualized with Odyssey infrared imaging system (Li-COR Lincoln, NE).

### Cell proliferation assay

Cellular proliferation was analyzed using cell counting kit-8 (CCK-8) (Beyotime, Nantong, China). Bcap-37 cells and MDA-MB-231 cells were seeded at a density of 2 × 10^3^ per well and 1 × 10^4^ per well in a 96-well culture plate. Cell proliferation was detected at 24, 48, 72, and 96 h, respectively. Then, 100 μl serum-free culture medium and 10 μl CCK-8 solutions were added to each well, followed by incubation at 37 °C for 1 h. The optical density at 450 nm was measured on an ELX-800 spectrometer reader (Bio-Tek Instruments, Winooski, USA).

### Cell-cycle analysis

After transfection, cells were harvested and washed with PBS three times. Then, the cells were fixed with 70% cold ethanol at −20 °C overnight. Cells were resuspended in 1 ml DNA staining solution and incubated for 30 min at room temperature. DNA content of cells was quantified by FACScan flow cytometer (BD Biosciences, San Jose, CA, USA). Data on cell cycle distribution were analyzed using ModFit LT 3.0 software.

### Measurement of intracellular calcium

The cytoplasmic calcium concentration in breast carcinoma cells was measured using the Ca^2+^-sensitive fluorescent dye Furo 3-AM (Dojindo Laboratories, Japan). After transfection, cells were washed with HBSS three times and incubated in 1000 μl HBSS containing 5 μl Fluo 3-AM for 1 h at 37 °C in the dark. Then, cells were washed with HBSS three times, harvested, and analyzed using a FACSCanto flow cytometer (BD Biosciences, San Jose, CA).

### Wound-healing assay

For wound-healing assay, cells were seeded into six-well plates in culture medium and were transfected with either siRNA or plasmids. The cells were starved with serum-free medium overnight and incubated with 10 μg/ml mitomycin C (Roche, Germany) for 2 h prior to the scratch assay. A scratch was drawn at the center of the well, and then the wounded monolayer was photographed at the indicated time via fluorescent microscopy (Ti-U, Nikon, Japan) with ×100 magnification. Wound-healing percentage of the cells was determined by the ratio of healing width at each time point to the wound width at 0 h.

### Migration and invasion assay

Cell migration and invasion assay were performed using modified two-chamber plates with pore size of 8 μm. For the migration assay, 2 ×10^4^ Bcap-37 and 1.5 ×10^4^ MDA-MB-231 cells were seeded in serum-free medium in the upper chamber. For the invasion assay, 3×10^4^ Bcap-37 and 4.5×10^4^ MDA-MB-231 cells were added to the top chamber coated with matrigel (BD Biosciences, NJ, USA). To stimulate migration or invasion, complete medium was added to the bottom wells. After 12 h or 24 h incubation at 37 °C, cells in the upper chamber were carefully removed with a cotton swab and the cells that had traversed the membrane were fixed in methanol and stained with Crystal Violet Staining Solution. For quantification, five fields (up, down, median, left, right ×200) per filter were counted under a microscope.

### Statistical analysis

Values are expressed as mean ± SD. All statistical analyses were carried out using SPSS 16.0 software (SPSS). Statistical analysis in treatment groups were assessed by Student’s t-tests. All experiments were performed at least three times unless otherwise indicated. *P*-value < 0.05 was considered statistically significant.

## Additional Information

**How to cite this article**: Di, J. *et al.* Rap2B promotes proliferation, migration, and invasion of human breast cancer through calcium-related ERK1/2 signaling pathway. *Sci. Rep.* **5**, 12363; doi: 10.1038/srep12363 (2015).
